# Difficult diagnosis of atypical kawasaki disease in an infant younger than six months: a case report

**DOI:** 10.1186/s13052-017-0345-0

**Published:** 2017-03-08

**Authors:** L. Petrarca, R. Nenna, P. Versacci, A. Frassanito, G. Cangiano, A. Nicolai, F. Scalercio, L. Lo Russo, P. Papoff, C. Moretti, F. Midulla

**Affiliations:** Department of Pediatrics, “Sapienza” University of Rome, V.le Regina Elena 324, 00161 Rome, Italy

**Keywords:** Kawasaki disease, Fever, Coronary aneurysm, Case report

## Abstract

**Background:**

Kawasaki disease (KD) is an acute inflammatory vasculitis of unknown origin.

**Case presentation:**

We report the case of a 5-month-old child with an atypical form of KD, characterized by undulating symptoms, who developed an aneurysm of the right coronary artery and an ectasia of the left anterior descending coronary artery.

**Conclusion:**

This case report underlines the difficulties in recognizing incomplete forms of the illness in young infants, who are at higher risk of cardiac complications.

## Background

Kawasaki disease (KD) is an acute inflammatory vasculitis of small- and medium-sized arteries that can cause coronary artery weakening, aneurysm development and myocardial infarction. The disease usually occurs in infants and young children [1]. Although one or multiple infectious triggers are most likely, the precise etiology is still unknown. Due to the absence of a specific diagnostic test or pathognomonic clinical feature, currently, the diagnosis of KD is still made on clinical criteria [2]. Atypical or incomplete forms of KD are common (15–20% of all patients) especially in children younger than 6 months [3] or older than 5 years, with a higher incidence of coronary artery abnormalities [4, 5], long term consequences [6] and resistance to therapy [7]. Therefore, a high index of suspicion is needed for any infant or child with protracted fever of unknown origin, in order to avoid a missed diagnosis. Here we report the case of a 5-month-old child with an atypical form of KD, characterized by undulating symptoms, who developed an aneurysm of the right coronary artery and an ectasia of the left anterior descending coronary artery.

## Case presentation

A full term delivered, previously healthy, 5-month-old boy presented at our Emergency Room Department with fever for a total of 4 days, diarrhea, cough and irritability. He was being treated with oral amoxicillin + clavulanic acid prescribed by the pediatrician for a left medium otitis diagnosed two days before. Fleeting hives was reported.

On physical examination, he presented good general condition, body temperature was 37.5 °C, no signs of dehydration (capillary refill time <2 s) and a reduction in air penetration in the lower lobes on chest auscultation. Normal physical examination of heart and abdomen. Blood tests showed a very high value of C-Reactive Protein (C-RP: 49.32 mg/dL); hemoglobin (Hb) was 8.8 g/dL, white blood cell (WBC) count was 11,080/mm^3^ with 79.3% neutrophils, and platelet (PLT) count was 429,000/mm^3^. Transaminases were normal, while gamma glutamyl transferase (GGT) was high (126 UI/L). Chest radiograph showed peribronchial cuffing and we started an additional therapy with oral clarithromycin at a dose of 15 mg/kg. Resin blood culture and serology for Epstein Barr Virus, Mycoplasma pneumoniae, Chlamydia pneumoniae, type 1 and 2 Herpes simplex virus, Parvovirus B19, Coxsackie viruses and Toxoplasma Gondii ruled out infective origin of symptoms. All serological tests were negative except for IgG anti-Cytomegalovirus. Abdominal ultrasonography showed a focal bowel wall thickening, mild edema around the gallbladder, mild hepatosplenomegaly and a lymphadenomegaly near the hepatic hilum.

After 3 days from admission, the infant continued to have fever and presented erythema involving the palms and the soles. Suspecting a KD for the presence of fever (>7 days), changes in extremities, history of rash and high C-RP levels, we requested a transthoracic echocardiography, which only showed a mild mitral valve regurgitation but no abnormalities of the coronary arteries.

On the fifth day of hospitalization the infant presented a subsidence of the fever and blood tests showed a reduction of C-RP (6.2 mg/dL), GGT (85 UI/mL) and Hb (7.3 g/dL), together with an increase in the WBC count (17,180/mm^3^) and platelet (594,000/mm^3^).

In order to rule out an early onset of inflammatory bowel disease, we performed fecal calprotectin and fecal occult blood, both tested negative, and a second abdominal ultrasonography, which showed an improvement of the picture (reduced bowel thickening and no edema around the gallbladder). Moreover, the infant had always fed willingly and his diarrhea had gradually resolved.

**Fig. 1:**
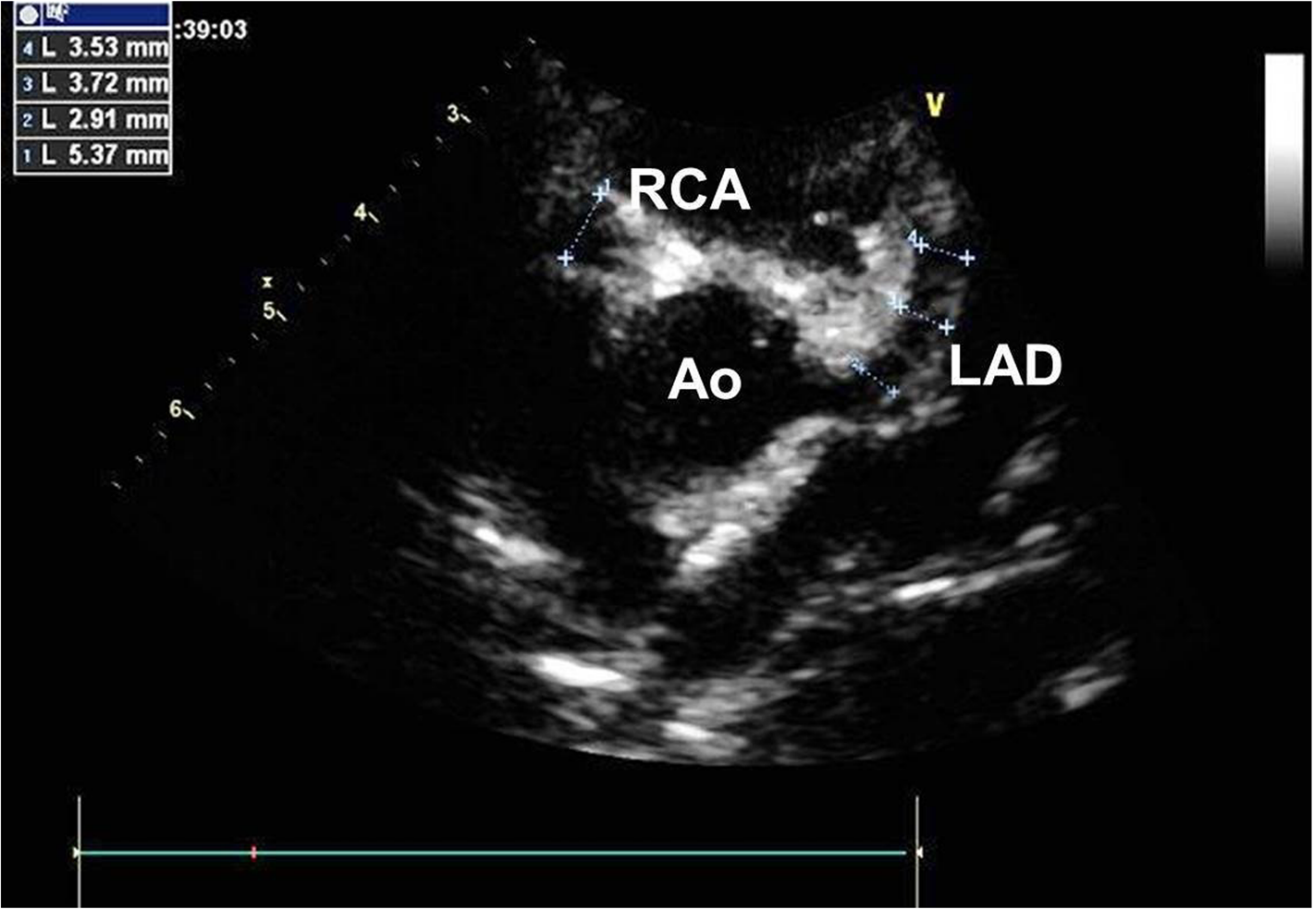
Transthoracic echocardiography of the patient showing the fusiform aneurysm of the right coronary artery and the ectatic left anterior descending coronary artery. Ao: aorta; RCA: right coronary artery; LAD: left anterior descending coronary artery

However, on the sixth day of hospitalization reappearance of the fever was registered (38.0 °C) and blood tests were performed on the eighth day showing an additional worsening of the anemia (Hb 7.2 g/dL), thrombocytosis (platelets 751,000/mm^3^), high WBCs (17,920/mm^3^) and an increase of C-RP (15.7 mg/dL). Serum iron, transferrin, lipid profile were within normal limits. We performed a second echocardiography, which revealed a fusiform aneurysm of the right coronary artery measuring 5.4 mm in diameter and an ectasia of the left anterior descending coronary artery, whose maximum diameter was 3.7 mm with a z-score +9.72 based on international standards [4] (Fig. 1). According to the American Heart Association (AHA) criteria the diagnosis of KD was made [2]. He received intravenous immunoglobulin (IVIG: one bolus of 2 g/kg) and high-dose oral acetylsalicylic acid (ASA) (100 mg/kg/day). However, after two days of persistent fever, a second bolus of IVIG was administered.

On the eleventh day of hospitalization, a severe anemia occurred: red blood cells (RBC) 2,660,000/mm^3^, Hb 5.9 mg/dl, Ht 19.8%, MCV 78 fL, RDW 15.2%. Bone marrow examination showed mild eosinophilia (5%), and a RBC transfusion was necessary. We reduced ASA dose to 5 mg/kg/day to achieve an anti-aggregant effect for the presence of a severe thrombocytosis (PLT 1,147,000/mm^3^).

Forty-eight hours after the second dose of IVIG, due to persisting fever, we started intravenous methylprednisolone at a dose of 30 mg/kg/day for three subsequent days, with initial benefit. However, after three days from the last administration of corticosteroid therapy we recorded a reappearance of the fever (38.0 °C) together, with a further dilatation of the left anterior descending coronary artery and we started oral prednisolone at a dose of 2 mg/kg/day for 30 days. Fever disappeared after 48 h.

The patient was discharged after 26 days of hospitalization, afebrile, with oral ASA. He is being regularly monitored to check the size of the coronary arteries. The last echocardiography performed 18 months after the KD onset showed a mild reduction of the aneurysm of the right coronary artery (3.6 mm) and of the ectasia of the left anterior descending coronary artery (2.5 mm, z-score +2.46). He is still on therapy with oral ASA.

## Discussion

KD is an autoimmune vasculitis of unknown etiology that occurs predominantly in infants and young children, with an age distribution ranging from 3 months to 10 years [1].

The classic diagnosis of KD is based on clinical evidence of prolonged fever (≥5 days) associated to the presence of at least four out of five principal clinical features (change in extremities, polymorphous exanthema, bilateral bulbar conjunctival injection without exudate, changes in lips and oral cavity, cervical lymphadenopathy) [2]. Typically, all of the clinical features are not present at a single point in time, as was the case with our young patient, in which the exanthema was present within the first two days of the onset of the fever.

Coronary artery aneurysms or ectasia develop in 15 to 25% of untreated children with the disease and may lead to myocardial infarction, sudden death, or ischemic heart disease [6].

In our case KD diagnosis was extremely difficult because the child presented an atypical manifestation of the disease. Albeit KD was considered one of the possible diagnosis we have been initially misled by the first echocardiography that showed no coronary artery alterations together with the disappearance of the fever for 36 h. We didn’t start the IVIG immediately because the patient presented only two out of five typical clinical features besides prolonged fever. According to the AHA guidelines (2) in this case, KD diagnosis can be made only if coronary abnormalities are present.

Our patient, after an initial defervescence, developed coronary artery aneurysm (currently still present), which was evident at the second echocardiography performed after 10 days from the onset of the fever, and showed an IVIG resistant disease.

This case report underlines the usefulness of guidelines in preventing overdiagnosis of KD, but also the difficulties in recognizing incomplete forms of the illness when following them, especially in very young infants, of less than six month of age, who are at higher risk of cardiac complications. Moreover, our case emphasizes the usefulness of ancillary laboratory findings (high C-RP, GGT and WBC, thrombocytosis, anemia) for suspected KD and, as reported in literature, high C-RP end GGT levels at presentation are significant predictors for IVIG resistant disease [7, 8].

## Conclusions

In conclusion, our case report suggests that KD should always considered in the diagnostic flow-chart of protracted fever of unknown origin, especially in very young children. Moreover, due to the fact that in children with incomplete KD, early echocardiography could be negative for specific signs of coronary disease, it is mandatory to perform frequently repeated exams during the hospitalization, in order to detect the most feared complication of the disease.

## Abbreviations

AHA: American heart association
ASA: Acetylsalicylic acid
C-RP: C-Reactive Protein
GGT: Gamma glutamyl transferase
Hb: Hemoglobin
IVIG: Intravenous immunoglobulin
KD: Kawasaki disease
PLT: Platelet
WBC: White blood cell
